# Horizontal Gene Transfer

**DOI:** 10.1093/emph/eov018

**Published:** 2015-07-29

**Authors:** Alita R. Burmeister

**Affiliations:** ^1^ Department of Microbiology and Molecular Genetics,; ^2^ Department of Ecology, Evolutionary Biology, and Behavior Program, and; ^3^ BEACON Center for the Study of Evolution in Action, Michigan State University

## DEFINITION AND BACKGROUND

Horizontal gene transfer (HGT) is the movement of genetic information between organisms, a process that includes the spread of antibiotic resistance genes among bacteria (except for those from parent to offspring), fueling pathogen evolution.

**Figure 1.**
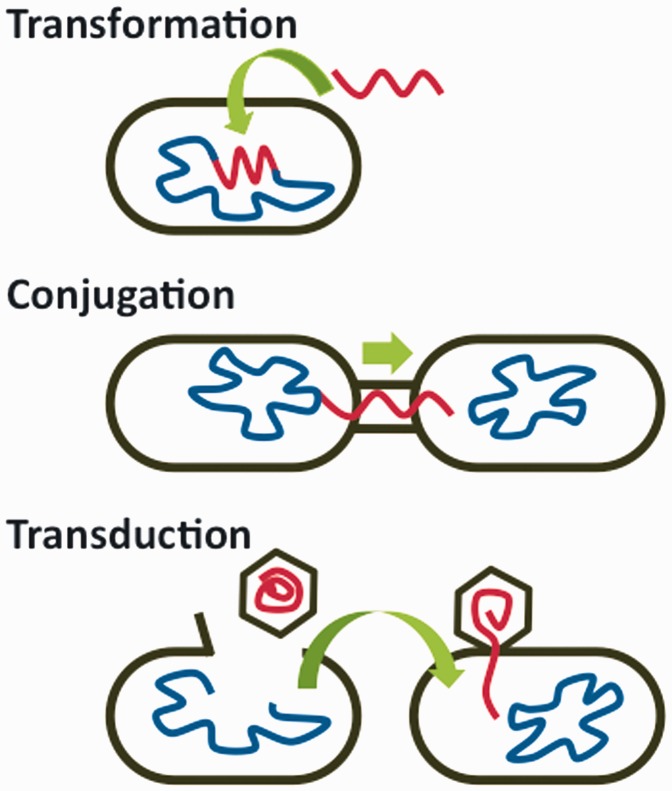
Mechanisms of bacterial horizontal gene transfer

Many resistance genes evolved long ago in natural environments with no anthropogenic influence but these genes are now rapidly spreading to and among human pathogens. HGT occurs by three well-understood genetic mechanisms (Fig. 1):

- Transformation: Bacteria take up DNA from their environment
- Conjugation: Bacteria directly transfer genes to another cell
- Transduction: Bacteriophages (bacterial viruses) move genes from one cell to another

Once transferred, the genes and pathogens continue to evolve, often resulting in bacteria with greater resistance [1, 2, 3, 4]. All genes—not just those causing drug resistance—may be horizontally transferred and proliferate by natural selection, including virulence determinants [5].

## EXAMPLES IN HUMAN BIOLOGY AND PUBLIC HEALTH

Antibiotic use in human medicine and agriculture continually selects for resistant bacteria [2, 6]. For example, tetracycline and β-lactams commonly fed to animals provide a selective environment for tetracycline and methicillin resistance. Genes conferring resistance to these antibiotics have horizontally transferred into a sensitive human-associated *Staphylococcus aureus* strain, resulting in methicillin-resistant strain CC398 [7]. After a strain gains resistance by HGT, the bacteria proliferate and continue to evolve as they move among patients and hospitals [1]. This process occurs in many bacterial lineages, resulting in diverse populations of a variety of strains, such as USA300 [5].

## EXAMPLES IN CLINICAL MEDICINE

Ongoing HGT poses a problem for clinical surveillance and treatment. Bacterial populations evolve rapidly, resulting in diversity that necessitates individual screening to determine effective treatments and to detect new strains, such as methicillin and high level vancomycin resistant *S.aureus* (MRSA and VRSA) [2]. Even when new drugs and diagnostic tools become available, the persistence of HGT will require ongoing surveillance for newly resistant pathogens, leaving practitioners and researchers racing with evolution.
